# Hyperspectral Computed Tomographic Imaging Spectroscopy of Vascular Oxygen Gradients in the Rabbit Retina *In Vivo*

**DOI:** 10.1371/journal.pone.0024482

**Published:** 2011-09-13

**Authors:** Amir H. Kashani, Erlinda Kirkman, Gabriel Martin, Mark S. Humayun

**Affiliations:** 1 Doheny Eye Institute, University of Southern California, Los Angeles, California, United States of America; 2 Department of Animal Resources and Veterinary Medicine, University of Southern California, Los Angeles, California, United States of America; 3 Reichert Technologies, Buffalo, New York, United States of America; 4 Department of Ophthalmology, Keck School of Medicine, University of Southern California, Los Angeles, California, United States of America; 5 Department of Biomedical Engineering, Viterbi School of Engineering, University of Southern California, Los Angeles, California, United States of America; University of Arizona, United States of America

## Abstract

Diagnosis of retinal vascular diseases depends on ophthalmoscopic findings that most often occur after severe visual loss (as in vein occlusions) or chronic changes that are irreversible (as in diabetic retinopathy). Despite recent advances, diagnostic imaging currently reveals very little about the vascular function and local oxygen delivery. One potentially useful measure of vascular function is measurement of hemoglobin oxygen content. In this paper, we demonstrate a novel method of accurately, rapidly and easily measuring oxygen saturation within retinal vessels using *in vivo* imaging spectroscopy. This method uses a commercially available fundus camera coupled to two-dimensional diffracting optics that scatter the incident light onto a focal plane array in a calibrated pattern. Computed tomographic algorithms are used to reconstruct the diffracted spectral patterns into wavelength components of the original image. In this paper the spectral components of oxy- and deoxyhemoglobin are analyzed from the vessels within the image. Up to 76 spectral measurements can be made in only a few milliseconds and used to quantify the oxygen saturation within the retinal vessels over a 10–15 degree field. The method described here can acquire 10-fold more spectral data in much less time than conventional oximetry systems (while utilizing the commonly accepted fundus camera platform). Application of this method to animal models of retinal vascular disease and clinical subjects will provide useful and novel information about retinal vascular disease and physiology.

## Introduction

Spectroscopy provides a potentially useful method of probing tissue structure and function in a non-invasive manner and has been applied in many fields including circulatory physiology [1], [2], oncology [3], [4], genomics [5], fluorescence microscopy [6], intravital microscopy [7], [8], environmental science [9], dermatology [10], and ophthalmology [11]. Most spectroscopy methods use filter-wheels (band sequential scanning) which limit temporal resolution, spectral range and confound spatial registration of data if the scene is not static. This is problematic for *in vivo* imaging since some degree of motion artifact is usually unavoidable. Current methods also have limited signal-to-noise ratios and are particularly susceptible to the confounding effects of pigmentation, optical media and scatter. One active area of imaging spectroscopy has been the study of hemoglobin oxygen saturation which is particularly relevant to the study of retinal vascular disease. Early investigators showed a difference between gross arterial and venous oxygen saturation using photographic emulsions and multiple filter systems [1], [2], [11], [12]–[14]. Subsequently, more sophisticated methods allowed near simultaneous measurements using a few wavelengths (usually between 2–4) to calculate gross oxygen saturation but these methods have not provided additional physiologically meaningful data over the earlier methods [15]–[22]. Most recently, the use of liquid-crystal tunable filters has allowed very rapid band-sequential spectral imaging but this system still requires image registration and up to15 minutes for data acquisition [16], [23]. These limitations have presented challenging problems for dual-wavelength and band-sequential spectral measurements. Nonetheless, *in vivo* application of these methods in human eyes suggests that, under some physiological and pathological conditions, there may be variations in retinal arteriovenous (AV) oxygen content [24]–[29]. Further study of these fluctuations at a microvascular level has not been reported due to the limited spectral range and signal-to-noise of current instruments.

Hyperspectral imaging is a generic term applied to any form of imaging in which dense sampling of the spectral information is achieved. This methodology has been widely applied in the astronomical and earth sciences but it has been only recently adapted biologically for rapid, high resolution, non-invasive imaging of microvasculature *in vivo* [26]. In the context of this paper, “hyperspectral” refers to the simultaneous (within 3 msec) acquisition of spectra from 450–700 nm with ∼4 nm spectral resolution using a two dimensional diffraction grating and computed tomographic imaging algorithms [30], [31]. We refer to this system as a “hyperspectral computed tomographic imaging spectrometer” (HCTIS).

In this paper, we implement the HCTIS to measure hemoglobin oxygen gradients within the microvasculature of the retina *in vivo*. We successfully demonstrate that the HCTIS can provide highly coregistered temporal, spatial and spectral data with sufficient resolution to allow visualization of intravascular oxygen gradients, in more detail than currently available systems. Further characterization of these gradients is likely to improve our understanding of retinal vascular disease and vascular physiology.

## Materials and Methods

### Animal Surgery and Procedures

**Figure 1:**
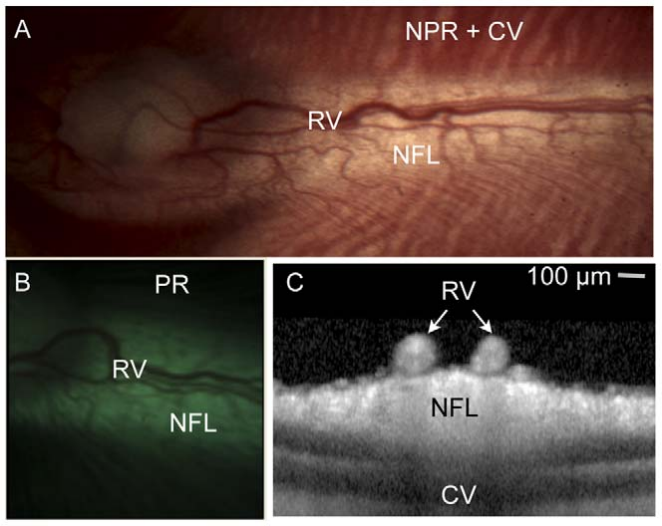
Illustration of rabbit retinal anatomy in one representative albino (panel a) and pigmented (panel b) animal. (A,B) Color photographs of the retina at low magnification illustrate the relationship between the retinal vessels (RV), non-pigmented nerve fiber layer (NFL), non-pigmented retina (NPR) with visible choroidal vessels (CV) and pigmented retina that obscures underlying choroidal vessels (PR). (C) Optical coherence tomography of the retina in cross-section demonstrates that the retinal vessels lay on top of the unpigmented, myelinated nerve fiber layer. Note the complete lack of pigmentation in the albino animal versus the pigmented animal. Scale bar 100 microns (for panel C only).

All protocols and procedures were approved by the University of Southern California Institutional Animal Care and Use Committee (IACUC approval number 11185) and are in accord with the Association for Research in Vision and Ophthalmology (ARVO) statement on the use of animals in ophthalmic and visual research as well as the Weatherall report. All procedures were performed under anesthesia and every attempt was made to minimize animal suffering. New Zealand pigmented and albino rabbits weighing 3–4 kg were used as an animal model for a number of reasons. First, it is well known that rabbits can tolerate relatively large fluctuations in systemic vitals such as heart rate, respiratory rate and hemoglobin oxygen saturation during anesthesia [32]–[33]. This physiology provides an excellent animal model to calibrate our spectral measurements over a wide range of *in vivo* hemoglobin oxygen saturation. In addition, the well described anatomy of retinal vessels provides an ideal imaging target *in vivo* [34]–[38]. Specifically, the largest retinal vessels (100–200 micron diameter) in rabbits lay on top of a non-pigmented nerve fiber layer (Figure 1). In addition, we used both pigmented and albino rabbits in our experiments to assess the potential effects of peripheral tissue pigmentation on our measurements (Table 1). Animals were sedated using standard intramuscular injections of ketamine (50–80 mg/kg) and xylazine (5–10 mg/kg). Pupils were dilated using 2.5% phenylephrine and 0.5% tropicamide ophthalmic solutions. Unless otherwise indicated, animals were sedated only with intramuscular or subcutaneous ketamine and xylazine as above without intubation and ventilation.

**Table 1:** Comparison of arterial and venous HypS_ox_ measurements and arteriovenous (AV) differences from 4 rabbits (2 albino and 2 pigmented).

Table 1	HypS_ox_		AV Difference	P-Value
	Artery	Vein		
Pigmented Rabbit 1a	87±9%	77±12%	10%	0.03
Pigmented Rabbit 1b	91±14%	80±7%	11%	0.04
Pigmented Rabbit 2	86±10%	90±9%	4%	0.14
Albino Rabbit 3	95±8%	87±8%	8%	0.04
Albino Rabbit 4	82±6%	78±4%	4%	0.18

First two rows (Pigmented Rabbit 1A and 1B) show data from two different parts of the same pigmented rabbit retina demonstrating the reproducibility of the measurement. All other measurements are from different rabbits. Peripheral retinal pigmentation did not significantly affect these measurements. P-values determined using paired, two-tailed, Student T-test.

### Hyperspectral Imaging

**Figure 2:**
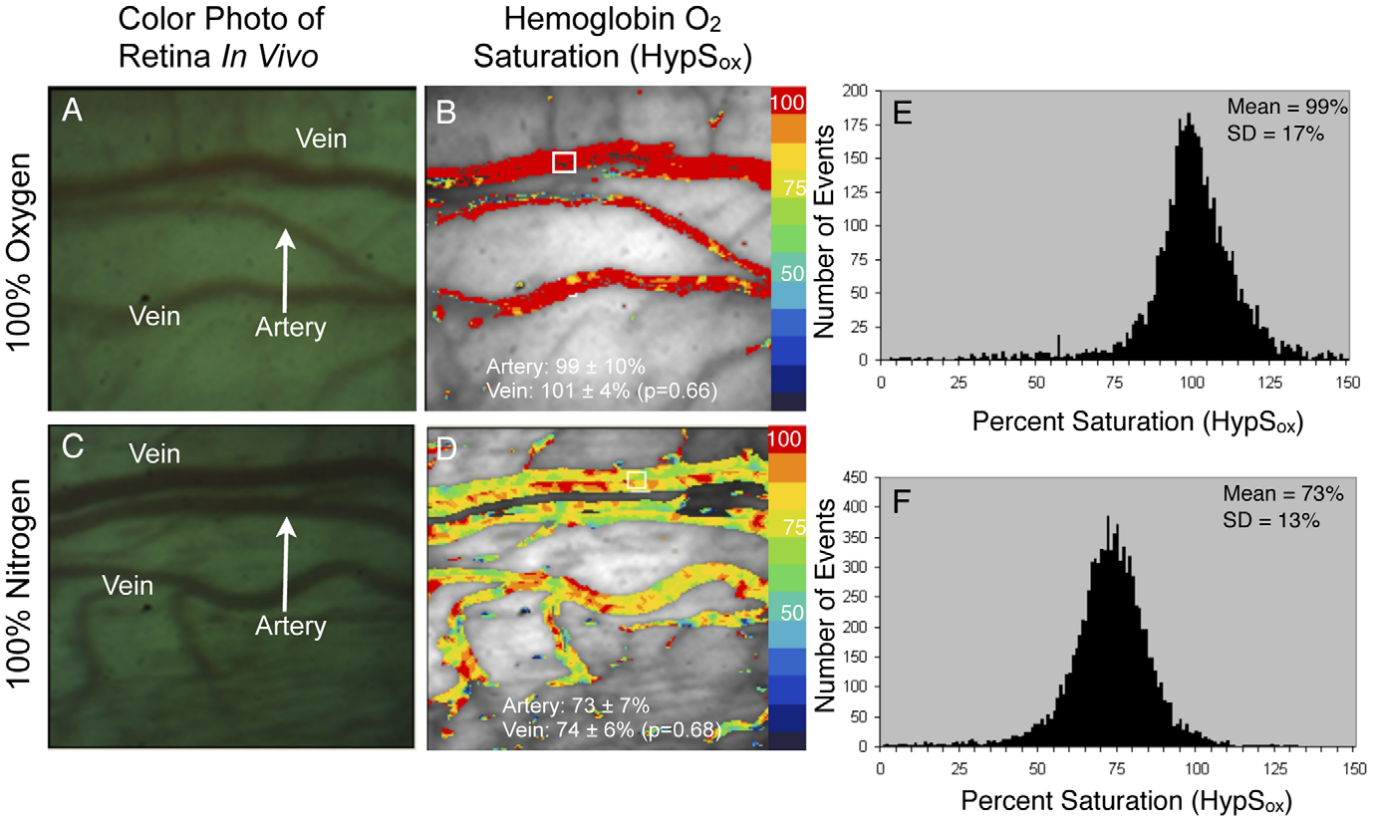
Hemoglobin oxygen saturation (HypS_ox_) in rabbit retinal vessels during artificial ventilation with oxygen and nitrogen. (A) Color image of retinal vessels while rabbit is ventilated with 100% oxygen and (C) same rabbit ventilated with 100% nitrogen. Images are approximately of the same area. (B,D) Pseudocolored maps of HypS_ox_. (B) HypS_ox_ was uniformly high for all vessels after ventilation with 100% oxygen (Mean artery = 99±10%, Mean vein = 101±4%; two-tailed, paired, Student T-test, p = 0.66, n = 10). (D) HypS_ox_ gradually decreased in all vessels during ventilation with 100% nitrogen gas (Mean artery = 73±7%, Mean vein = 74±6%; two-tailed, paired, Student T-test, p = 0.68, n = 10). Arterial and venous HypS_ox_ was calculated by taking the average of 10 non-overlapping measurements along the artery and vein (small white box in panel B illustrates one such measurement area). (E,F) Histograms illustrating the approximately Gaussian distribution of HypS_ox_ measurements for all pixels in panel 2D and 2B. Since arterial saturation cannot be greater than 100% based on blood gas measurements, all pixels with saturation values greater than 100% were scaled to red in the corresponding images. The Student T-test was used to compare the HypS_ox_ of the artery and vein. Color bar represents the percent saturation of hemoglobin with red = 100% and blue = 0%.

**Figure 3:**
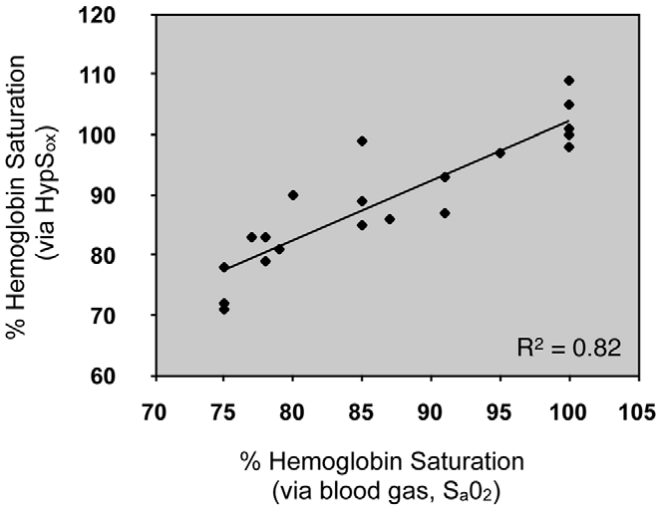
Correlation of arterial blood gas (S_a_0_2_) and HypS_ox_ measurements in the rabbit. Correlation of S_a_0_2_ from the auricular artery and HypS_ox_ from the retinal artery (n = 4 rabbits; multiple measurements were made in all rabbits). The solid line represents the best fit linear regression. R^2^ represents the square of the Pearson correlation coefficient.

**Figure 4:**
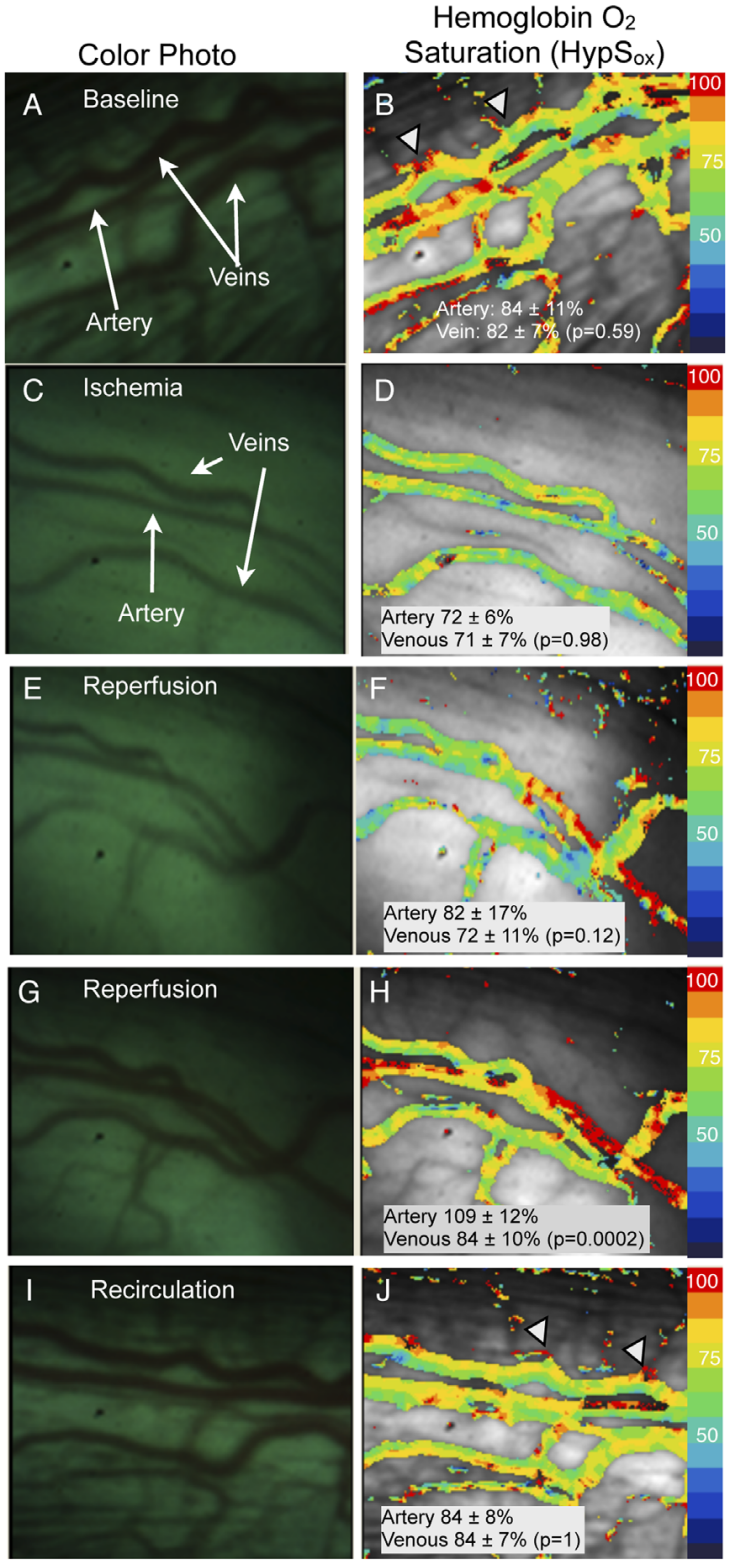
Changes in HypS_ox_ gradients during ischemia and subsequent reperfusion of retinal vessels. Color fundus images of retinal vessels (panels A,C,E,G,I) with corresponding pseudocolored HypS_ox_ measurements (panels B,D,F,H,J). (B) HypS_ox_ measurements at baseline and subsequently (D) after elevating intraocular pressure (IOP) to induce ischemia. Note significant desaturation of arterial and venous HypS_ox_ post-ischemia (Artery: 84±11% versus 72±6%, p = 0.017; Venous: 82±7% versus 72±7, p = 0.00006; paired, two-tailed Student T-test, n = 10). The third row (E,F) and fourth row (G,H) panels correspond to images acquired in succession during reperfusion. Arterial saturation values increase from 72±6% (panel D) to 109±12% (panel H) as the artery is reperfused (n = 10, p = 0.00001, paired, two-tailed Student T-test). The last row (panel I,J) illustrates HypS_ox_ measurements after 15 minutes of reperfusion (i.e. recirculation). Notice the prominent intravascular oxygen gradients when comparing panel 4B (ischemia) and panel 4J (recirculation). Images may not show exactly the same field-of-view and each image may vary slightly in orientation due to ocular movements.

Imaging was performed through a dilated pupil with a custom-made hyperspectral camera attached to the top port of a standard, commercially available Zeiss FF450 IR fundus camera. The hyperspectral computed tomographic imaging spectrometer (HCTIS) has been previously described [29]. Briefly, the HCTIS captures spatial and spectral information by imaging a scene through a two-dimensional grating which produces multiple spectrally dispersed images of the retina onto a commercially available focal plane array (Retiga 2000R Camera; QImaging Inc, BC, Canada) [29]. The dispersion pattern of spectra on the focal plane array is calibrated in advance with a monochromator. Images are recorded by a digital camera and stored on a computer using standard image acquisition software (QImaging Inc; BC, Canada). The camera acquires approximately 76 spectral bands in approximately 3 msec (standard fundus flash illumination) over the range 450–700 nm [29]. Consequently, hyperspectral images are not blurred by microsaccades and do not need image registration to eliminate motion artifact or pixel misregistration. There are no moving parts in this device and there is no need for spatial or spectral scanning since all the spectra are collected within the duration of a single flash photograph [29]. Therefore all spectra are spatially and temporally coregistered on the focal plane array. Custom made computed tomographic imaging algorithms based on iterative expected-maximization algorithms [30], [31] are used to reconstruct the spectrally dispersed images into a three-dimensional map of spatial (X-Y axis) and spectral (Z-axis) information that can be probed for individual wavelength information. Such a representation is sometimes called a “hyperspectral cube.” For each image, a “zero order” color image that is not dispersed or reconstructed is also captured [29]. This image is the equivalent of a standard fundus photo but dimmer and smaller in size due to the diffraction of light for spectral analysis. This image is provided in the figures for easy reference to the anatomy (Figures 2, 3 and 4). Authors who would like to access the data should feel free to contact the communicating author for data sets of interest. The camera and software are unique prototypes and therefore not commercially available. Authors who are interested in making use of the camera and/or software may feel free to reference the original paper describing the device [29] or contact Reichert Technologies.

### Ischemia Reperfusion Model

**Figure 5:**
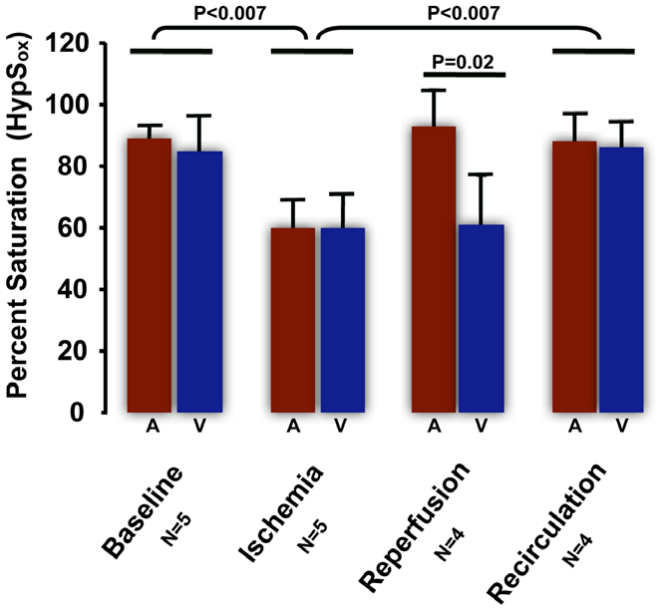
Summary results from 5 ischemia-reperfusion experiments (as described in **Figure 4**) with average arterial (A) and venous (V) HypS_ox_ measurements for each condition. Comparisons are made with Student T-test as shown.

In order to demonstrate the spectral, spatial and temporal resolution of the HypS_ox_ measurements we used an acute ischemia-reperfusion model to generate intravascular gradients (Figure 4 and 5). In these experiments, baseline HypS_ox_ measurements were made while the animal was sedated and breathing room air. Subsequently, an intraocular cannula was sutured into the *pars plana* space and attached to an elevated reservoir of balanced salt solution to increase intraocular pressure (IOP). This manipulation reliably caused an ischemic compartment syndrome of the retina by increasing IOP to approximately systolic blood pressure and preventing any retinal perfusion. IOP was measured using a Tonopen (Reichert Technologies Inc., Buffalo, NY). IOP was elevated (>90 mm Hg) by this method until the smallest retinal vessels were visibly blanched when viewed through the fundus camera and clinically by 20D indirect ophthalmoscopy. Under these circumstances, the largest retinal vessels remained visibly filled with a static column of blood which allowed subsequent HypS_ox_ measurements. It is important to note that the intraocular contents were not replaced by the infusion solution since no vitrectomy was performed and no appreciable fluid was lost from the saline reservoir during the entire procedure. In addition, there was no flow of saline solution as demonstrated by the lack of fluid movement in the IV drip chamber. Blood gas measurements (IStat; Abaxis Inc) were obtained from the auricular artery and vein 10–15 minutes after sedation and as needed thereafter. Arterial and venous blood gas measurements were made within 5 minutes of each other. Animals were sacrificed using standard intracardiac or intravenous injection of 120 mg/kg sodium pentobarbitol to induce cardiopulmonary arrest.

### Optical Coherence Tomography (OCT)

OCTs were performed on a Heidelberg Spectralis™ SD-OCT (Heidelberg, Germany) using dense raster scan patterns. Individual scans were viewed and edited using the commercially available software from the manufacturer (Figure 1).

### Hyperspectral Hemoglobin Oxygen Saturation (HypS_ox_) Measurement

The calculation of hemoglobin-oxygen saturation is based on a modified Lambert-Beer relationship of light reflected from within and adjacent to a vessel as described below. Multiple pixels are used in this measurement to achieve reliable and reproducible measurements. In our calculation of hemoglobin oxygen saturation, the apparent optical density, D_v_, of a retinal vessel is calculated by division of the signal intensity from within the vessel by the signal intensity from outside the vessel. The resulting ratio is the apparent transmittance, T_v_, of the vessel
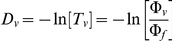
Where Φ_f_ represents the light reflected by the retina without traversing the vessel, and Φ_v_ represents the light that passes through the center of the vessel. Applying the Lambert-Beer law yields an equivalent mathematical expression of optical density that is weighted for the respective hemoglobin species:
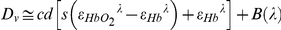
where c = c_HbO2_+c_Hb_ (the total concentration of hemoglobin), and s = (c_HbO2_)/(c_HbO2_+c_Hb_) or the oxygen saturation. ε_HbO2_ and ε_Hb_ are the extinction coefficients for oxy- and deoxyhemoglobin respectively [39]. “d” is the width of the blood vessel, and D_v_ is the optical density. Here, *B(λ)*, represents an undefined and complex term for the multiple sources of light scattering that occur from non-hemoglobin sources (surrounding tissue). Previous investigators have modeled this term using a constant due to limited spectral bandwidth [11], [15], [18], [19], [20]. For the present calculation we will ignore *B(λ)* and assume the spectral noise is negligible. Quantification of the *B(λ)* term is described later in the section entitled, “Spectral Noise Analysis.” The current model uses 28 empirically selected wavelengths from the hyperspectral cube to calculate *cd* and *cds* by means of a least squares approximation. From the equation for the optical density, we rewrite the equation for the optical density of each wavelength, λ_i_, as
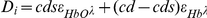
Let x ≡ cds and y ≡ cd - cds. The equation can then be rewritten:
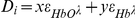
A least-squares approximation can be performed based on 28 wavelengths to find the unknown variables x and y. Subsequently, calculation of oxygen saturation, “s,” (oximetry) is possible. Oximetry calculations were applied to images using an unsupervised vessel masking algorithm and are displayed as pseudocolor images in which red represents 100% saturation and blue colors represent successively more desaturated hemoglobin. Oxygen saturation was calibrated using measurements obtained while rabbits were intubated and ventilated with 100% oxygen or 100% nitrogen as described above. Local oxygen saturation values within a vessel (artery or vein) were obtained by averaging saturation values from 10 separate, non-overlapping vessel segments each measuring 10×10 pixels (one such sample region shown as a small white box in Figure 2b). Comparisons between arterial and venous saturation were made using the average of 10 such non-overlapping measurements in each vessel.

### *In Vivo* Calibration

In order to calibrate the hyperspectral measurements of hemoglobin oxygen saturation (HypS_ox_) *in vivo*, some animals (n = 4; Figure 2 and 3) were intubated and ventilated with either 100% oxygen gas or 100% nitrogen gas using a Narcomed Ventilator while sedation was maintained with Sevofluorane. Blood pressure, temperature, pulse oximetry, pO_2_, pCO_2_, pH, HCO_3_ and SaO_2_ were also monitored. In these experiments, HypS_ox_ measurements were made from a dilated eye while the animal was ventilated with the indicated gas. Reference blood gas measurements were made from the auricular artery using an IStat ABG kit (Abaxis Inc.) for comparison with HypS_ox_ measurements from the retinal vessels at a given blood oxygen saturation. The blood gas measurements agreed well with peripheral pulse oximetry measurements from the paw but only blood gas measurements were used for analysis. During ventilation with 100% oxygen, the arterial blood gas measurement (from the auricular artery) was used as the standard for 100% oxygen saturation because it was most likely to be stable throughout the body circulation (venous circulation would likely reflect differences in local tissue oxygen metabolism which could vary dramatically at different body sites).

### Spectral Noise Analysis

As mentioned above, the complete equation describing the optical density of a vessel includes B(λ) which represents an undefined and complex term for the multiple sources of light scattering that occur from non-hemoglobin sources (surrounding tissue). These aberrant reflections and sources of scatter contribute artifactual signals to the measurement of the apparent optical density at any given pixel and are collectively termed “spectral noise.” Although it is not possible to eliminate the spectral noise in our analyses, we are able to quantify the magnitude of the noise for any given pixel based on the difference between the *calculated* optical density from our least-squares optical density approximation and the *measured* optical density from the raw images. This empirically quantitates the magnitude of the scattering term, B(λ), and is an estimation of all the sources of spectral noise within the image at each pixel. Unlike previous models, this error term is uniquely calculated for each pixel in each image and likely represents the most accurate estimation of spectral noise. To quantitate the amount of spectral noise in our images we define the spectral noise,▪, as
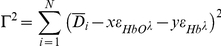
where D_i_ is the measured optical density from our images, and N is the number of wavelengths measured. Applying 
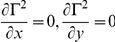
 we find:
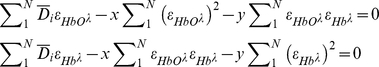
Then, we calculate x and y by solving the system of equations that represents the calculated optical density expected from the model without the scattering term B(λ).
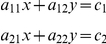
In this model, the spectral noise index (SNI) represents the average difference between the measured optical density values and calculated optical density values (based on the least squares approximation) for 28 wavelengths. In this model the 28 wavelengths were empirically selected within the range commonly used by investigators to study hemoglobin spectra (500–620 nm). Theoretically, if spectral noise did not exist and 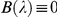 this difference should be zero. Spectral noise measurements are displayed as separate pseudocolored maps of noise intensity over the image of reference. In general, the spectral noise index was about 5% for most images.

### Statistics

The distribution of HypS_ox_ measurements was approximately Gaussian in the illustrated cases (Figure 2E, F). Therefore pair-wise comparisons were made using Student T-test (paired, two-tailed parameters). Correlation between peripheral hemoglobin oxygen saturation by arterial blood gas analysis and HypS_ox_ was performed using Pearson-Correlation coefficient function on Excel (Microsoft Inc., Seatte, WA). Bland-Altman graphs and Intraclass correlation coefficient (ICC) calculations were performed using SAS 9.13 programming language (SAS Institute, Cary, North Carolina, USA).

## Results

### *In Vivo* Calibration of Hyperspectral Hemoglobin Oxygen Saturation (HypS_ox_)

Figure 2 shows representative images and HypS_ox_ from rabbit retinal vessels *in vivo* after 10 minutes of ventilation with 100% oxygen (Figs. 2A,B) or 100% nitrogen (Figs. 2C,D). These conditions were used to calibrate *in vivo* HypS_ox_ measurements with peripheral oxygen saturation measurements obtained from the auricular artery (by blood gas). Ventilation with 100% oxygen resulted in a mean retinal arterial and venous oxygen saturation of 99±10% and 101±4% respectively (Fig. 2B). There was no significant difference between arterial and venous saturation under these conditions (paired, two-tailed, Student T-test, p = 0.66, n = 10). Arterial and venous HypS_ox_ measurements were made by averaging 10 measurements from non-overlapping 10×10 pixel regions (see example small white box in Fig. 2B) along the retinal artery and vein segments shown. Similar measurements were made during ventilation with 100% nitrogen gas as systemic arterial blood gas (ABG) S_a_O_2_ was allowed to decrease to between 70–80%. HypS_ox_ measurements under these conditions show a desaturation of the retinal vessels with a mean arterial oxygen saturation of 73±7% and venous saturation of 74±6%. There was no significant difference between arterial and venous saturation under these conditions (paired, two-tailed, Student T-test, p = 0.68, n = 10). However, the overall decrease in retinal arterial and venous saturation was statistically significant between 100% oxygen ventilation and 100% nitrogen ventilation conditions (retinal arteries 99±10% vs 73±7%; p = 0.00007 and retinal veins 101±4% vs 74±6%; p = 0.000001; paired, two-tailed, Student T-test, n = 10). To determine whether HypS_ox_ measurements were physiologically accurate, we correlated these measurements with gold-standard ABG measurements from the peripheral auricular artery. Figure 3 shows the correlation between peripheral ABG based measurements from the auricular artery and HypS_ox_ measurements from the retinal artery (R^2^ = 0.82; n = 4 rabbits). Overall, there was very good correlation between HypS_ox_ measurements and peripheral ABG measurements, verifying the accuracy of our spectral measurements and oximetry algorithms.

### Demonstration of Intravascular and Arteriovenous (AV) Oxygen Gradients

Figure 4A and 4B show examples of color fundus images and corresponding HypS_ox_ measurements from one representative normal retina while the rabbit was sedated and breathing room air. Under this condition, there appears to be gradients of hemoglobin oxygen saturation within individual retinal vessels as demonstrated by the non-uniform pseudocolor maps. These gradients appear largest at the confluence of small (<50 microns diameter) and larger vessels (100–200 microns diameter; arrowheads Fig. 4B and 4J). Average retinal arterial and venous HypS_ox_ from four rabbits are summarized in Table 1. The AV difference ranged from 4–11% and was variably significant regardless of the presence of peripheral retinal pigmentation (albino versus pigmented). These measurements in the rabbit are consistent with previous findings of dramatic fluctuations in arterial oxygen saturation with anesthesia [32], [33] and low AV differences in this animal [10].

To independently determine if the low AV difference measured in the retinal vessels was an artifact or possibly a result of anesthesia, we measured AV differences between the auricular artery and vein under similar conditions in 6 additional rabbits. Blood gas measurements from the artery and vein were usually made within 2–3 minutes of each other and always <5 minutes of each other. These measurements demonstrated an average AV S_a_0_2_ difference between the auricular artery and vein of 4±1.4% (AV pO_2_ difference = 4.5±2.1 mm Hg and AV pCO_2_ difference = 2.2±1.2 mm Hg (n = 6 rabbits)). These low AV differences in the peripheral blood samples support the HypS_ox_ findings and suggest that the low AV difference is a systemic condition that is likely a result of anesthesia [32], [33]. Since it is not possible to perform the imaging experiment with the rabbit unsedated, we are not able to determine the true AV difference in the rabbit without anesthesia.

### Ischemia-Reperfusion Animal Model

The lack of an AV difference noted above may be partially an artifact of insufficient spectral or spatial resolution in our method. To empirically demonstrate whether the spatial and spectral resolution of the HCTIS was sufficient to resolve intravascular hemoglobin oxygen gradients, we utilized an *in vivo* ischemia-reperfusion model to carefully generate intravascular oxygen gradients that we could then attempt to measure. In these experiments, the intraocular pressure was artificially raised (>90 mm Hg) by placement of an intraocular canula attached to an elevated saline reservoir. Under these conditions, substantial blanching of the smaller retinal vessels was noted within minutes indicating vascular stasis and ischemia (Fig. 4C). In addition, the hemoglobin remaining in the blood vessels is static and should gradually equilibrate with the surrounding tissue oxygen. Figure 4C and 4D illustrate the results of this experiment. HypS_ox_ measurements under ischemic conditions revealed a gradual desaturation of hemoglobin within a few minutes that stabilized after approximately 30 minutes (Figure 4B versus Figure 4D). The intravascular hemoglobin oxygen gradients noted at baseline were substantially reduced after 30 minutes of ischemia suggesting diffusion-limited equilibrium of the oxygen between the blood and surroundings. Notably, there were no visible collateral vessels feeding the larger vessels under these conditions. Return of intraocular pressure to baseline demonstrated a significant gradient of oxygenated hemoglobin flowing into the arteries and veins (Figure 4E–J). Oximetry measurements after 15 minutes of reperfusion revealed that intravascular hemoglobin oxygen gradients returned to baseline (Figure 4B and 4J). Similar results were repeated and confirmed in 5 other rabbits and summarized in Figure 5.

**Figure 6:**
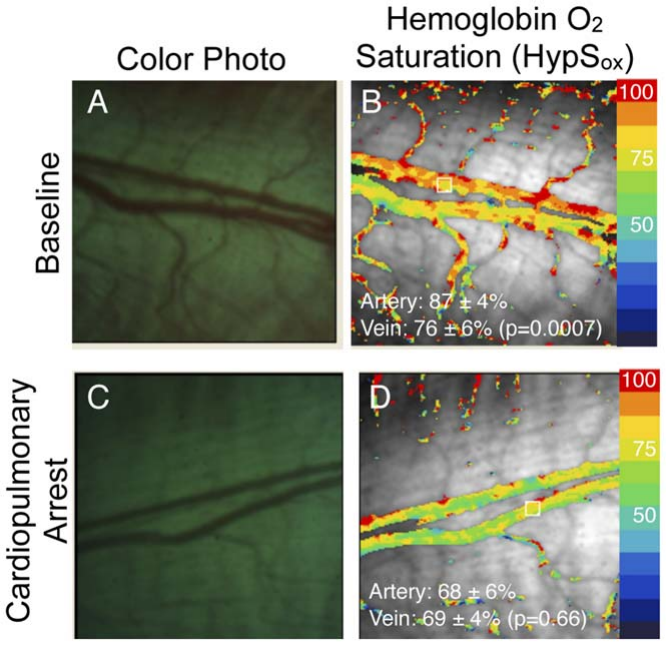
Hemoglobin oxygen saturation (HypS_ox_) measurement before (A,B) and after (C,D) cardiopulmonary arrest. HypS_ox_ measurements show significant desaturation of retinal artery and vein HypS_ox_ within 15 minutes of cardiopulmonary arrest. Comparisons between artery and vein were made using paired, two-tailed Student T-test. Desaturation between arterial and venous measurements was significant between panels B and D (Artery: 87±4% versus 68±6%; p = 0.00002, n = 10; Vein: 76±6% versus 69±4%, p = 0.03, n = 10; paired, two-tailed Student T-test).

The ischemia-reperfusion experiments in Figure 4 and 5 suggest that there are measurable gradients of hemoglobin oxygen saturation within retinal blood vessels, especially at the confluence of smaller and larger vessels, as well as between vessels. In addition, it suggests that the HCTIS has the capability to resolve these gradients. To further demonstrate that these gradients are physiological in nature and not an artifact of the system or our experimental manipulations, we performed one additional simple experiment. In one additional rabbit, we performed HypS_ox_ measurements in the same eye before and after cardiopulmonary arrest. In this case we observed that intravascular and arteriovenous differences in hemoglobin oxygen saturation were substantially attenuated after cardiopulmonary arrest (Figure 6). The presence of measurable variations in hemoglobin oxygen content before and after ischemia and cardiopulmonary arrest strongly suggests that the observed variations in HypS_ox_ measurements are largely a reflection of physiological changes in hemoglobin oxygen content among vessels feeding or draining surrounding tissues. In addition, the lack of such gradients in the 100% oxygen breathing state (Figure 2) further suggests that the origin of these gradients is partly physiological.

### Spectral Noise Analysis

**Figure 7:**
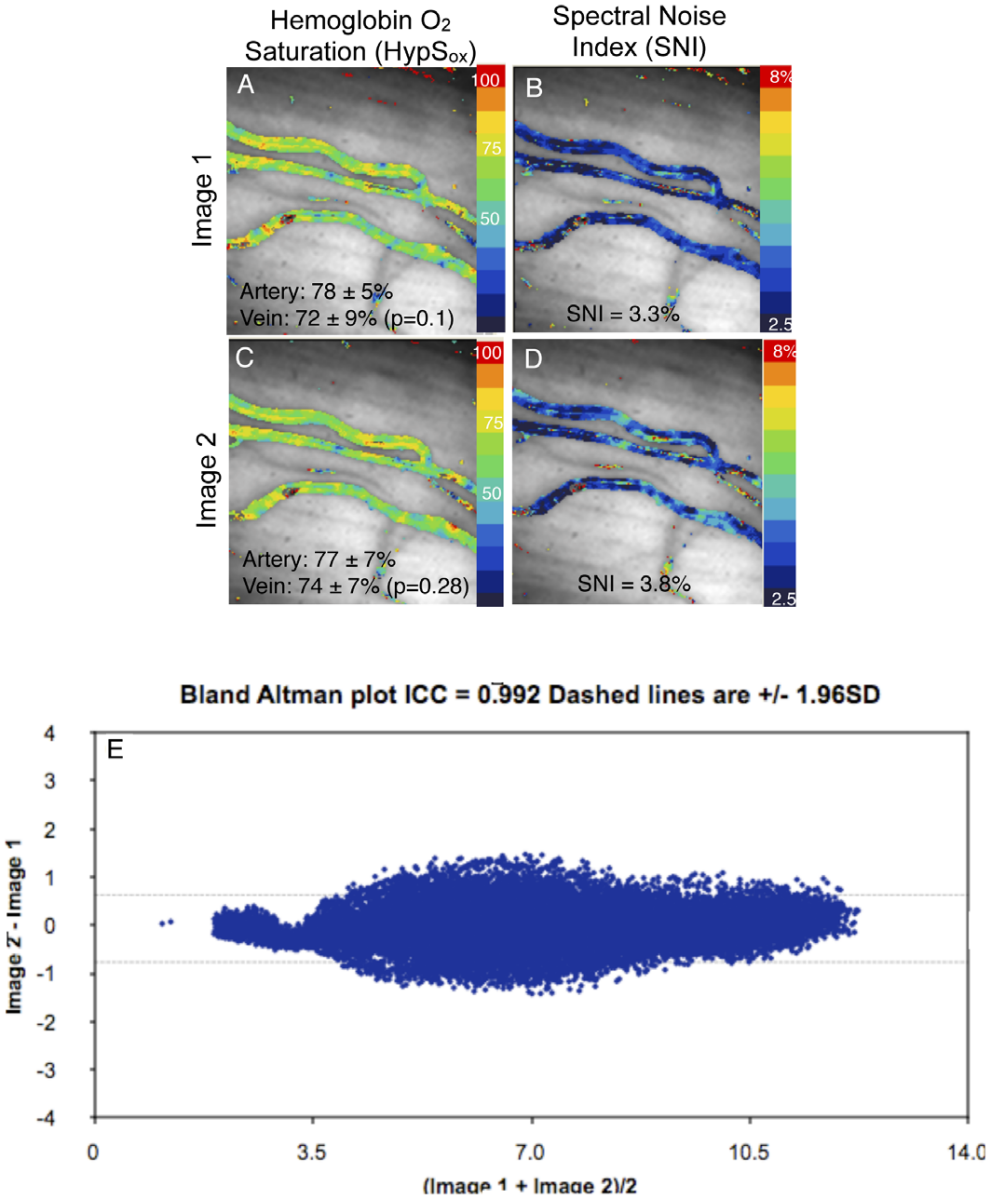
Spectral Noise Analysis. Two separate hemoglobin oxygen saturation measurements (HypS_ox_) from the same scene within one second intervals during ischemia (A,C). Sequential images were acquired during the ischemia phase of the experiment in Figure 4. Although there are very small changes in the qualitative patterns of hemoglobin oxygen saturation between the two images there is no difference in the average retinal arterial saturation or venous saturation between images (Artery: 78±5% versus 77±7%, p = 0.84, n = 10; Vein: 72±9% versus 74±7%, p = 0.30, n = 10, paired, two-tailed Student T-test) or within images (see panels for within image comparison). (B,D) Spectral noise maps were made from spectral noise index (SNI) calculations as described in the methods section. Noise maps illustrate the average percent variability for all wavelength intensities from one pixel to another. The SNI is an average of the variability for the whole image. (E) Pixel-to-pixel correlation of wavelength intensity for representative wavelengths demonstrates excellent correlation between two independent measurements of the same scene (ICC 0.992).

To quantitatively evaluate the contribution of spectral noise to the HypS_ox_ measurements, we performed additional analyses under conditions in which oxygen gradients within and between vessels would be insignificant. Figure 7 illustrates two images from the same scene that were obtained within a one second interval (same rabbit and target scene as in Figure 4D). Under these conditions of ischemia, there was no significant difference between HypS_ox_ measurements between the images (see figure legend). In addition, the random variation within each set of HypS_ox_ measurements was not significantly different using an F-test for equality of variance (p-value = 0.97 for pairwise comparison of oxygen distributions in panel 7A and 7C). Both of these findings suggests that random spectral noise was not a significant contributor to the observed intravascular gradients demonstrated above. One additional measure of random spectral noise is the variation in camera pixel intensity between any two images. With a significant degree of spectral noise, we would expect to see a substantial variation between the raw intensity of the same pixel for two images of the same scene. This measure would be independent of our algorithms for calculating HypS_ox_. Pixel-to-pixel correlation of spectral intensity between Figure 7A and 7C revealed an excellent correlation with Bland-Altman analysis (Fig. 7E; Intraclass correlation coefficient 0.992) further supporting the lack of large spectral variations due to random spectral noise.

In order to further assess the variability in our HypS_ox_ measurements in more a rigorous way, we devised a spectral noise index (SNI) to quantitate the amount of spectral variation from each pixel in our data set. The SNI quantitates the difference between optical density measurements made directly from the raw images and the optical density that is calculated by using the least-squares model described in the Materials and Methods section under “Hyperspectral Hemoglobin Oxygen Saturation Calculation (HypS_ox_).” Therefore, this measure should be zero if there is no scattering or diffraction of light by optical media, erythrocytes, pigmentation etc (i.e. no spectral noise). Since there are multiple sources of spectral noise that are not individually quantifiable in an *in vivo* system such as ours, the SNI is always a positive number and serves as an approximation of the “B(λ)” error term in our model of optical density. This is an empiric measure of the spectral noise that previous investigators have also quantified in different ways [7], [11], [14], [15], [16]. Unlike previous methods however, the large bandwidth of our hyperspectral device allows us to quantitate this term for every pixel in each image in a wavelength dependent manner without the need for repeated or multiple separate calibrations. Although this is a simple method of quantifying all the sources of spectral noise collectively (rather than attempting to quantify the contribution of spectral noise from every possible source such as pigmentation, media opacity, scatter etc), it is very practical for *in vivo* measurements since all the possible sources of spectral noise can vary dramatically. SNI measurements are displayed as a separate pseudocolored map of noise intensity for representative images (Figs. 7B and 7D). In general, the SNI was <5% for our images suggesting that the spectral noise contribution to HypS_ox_ measurements at any given pixel is about 5% of the displayed value.

## Discussion

We demonstrate proof-of-principal application of a rapid, completely non-invasive, and feasible method of hyperspectral tissue analysis. This method provides an excellent spectral, spatial and temporal coregistration *in vivo* compared to devices that rely on spectral or spatial scanning. This hyperspectral system is mounted on a commercially available fundus camera and demonstrates reliable and reproducible high resolution measurements of hemoglobin oxygen saturation (HypS_ox_) within retinal microvasculature *in vivo*. Using the hyperspectral data set, we develop and validate spectroscopic measurements of hemoglobin oxygen saturation and a spectral noise index (SNI). These measurements are resolved over a few milliseconds of time (standard flash photography) and within vessels as small as ∼50 microns wide. Using this tool, we show that rabbit retinal vessels can have a very low AV gradient under a range of inspired oxygen concentrations with standard anesthesia (75–100%). This is an important consideration for any investigator who choses the rabbit as a model system and may apply to other systems. We also observe gradients of hemoglobin oxygen saturation within individual vessels that have not been directly demonstrated previously due to limitations in the spectral, spatial and temporal resolution and coregistration. The strong correlation of our HypS_ox_ measurements with systemic measurements of oxygen saturation (ABG) shows that our unsupervised algorithms are accurate (Figure 3).

We demonstrate prominent intravascular retinal oxygen gradients that have not been previously reported *in vivo*. These intravascular gradients may represent physiological fluctuations in moment-to-moment hemoglobin saturation that result from mixing of blood within vessels. Similar findings have been observed during intravital microscopy of mucosal microvasculature in rodents [7], [8]. In some vessel segments, these gradients take on the appearance of laminar patterns consistent with laminar flow in vessels. The presence of these gradients is often coincident with the confluence of small and large vessel segments suggesting that vessels feeding or draining different tissues likely carry hemoglobin with substantially different saturation. The presence of gradients in apparently random locations may correlate with feeding vessels that merge from below the plane of the image and are not visible. Anatomical studies have demonstrated deep penetrating vessels within the thick nerve fiber layer of the rabbit [34]–[38]. The intravascular gradients may be a reflection of variations in local metabolic activity and represent a novel method of studying microvascular oxygen transport completely non-invasively.

We had to consider the possibility that the hemoglobin oxygen gradients may be an artifact of spectral noise from many potential sources including pigmentation, ocular media, blood flow, tissue scatter etc. This is unlikely for a number of reasons. First, within the same animal, these intravascular gradients are largely dampened under conditions of vascular stasis (ischemia), cardiopulmonary arrest or systemic hyperoxygenation (Figure 2,4,5,6). Furthermore, the largest gradients are often seen at the confluence of vessels where large differences in hemoglobin oxygen saturation could naturally occur. All these conditions are known to affect hemoglobin oxygen saturation in a logical and predictable way and our findings support those predictions. Second, pixel-to-pixel correlation of spectral intensity demonstrates an excellent correlation between two images of the same scene (Figure 7). Third, our independent measure of spectral noise (SNI) suggests an average spectral variability on the order of 5% of measured spectral intensity (Figure 7). It is important to keep in mind that our measurements are based on the representation of hemoglobin spectra over a few milliseconds and therefore represent a snapshot of the local oxygen saturation, not an average.

The intravascular gradients we have described may have not been recognized or demonstrated previously for multiple reasons. First, our study is in rabbits and other studies have been performed in different species. The only other study of rabbit retinal oximetry was conducted in 1975 using photographic emulsions and does not have sufficient spatial or spectral resolution to identify such gradients [14]. It is possible that intravascular gradients are specific to rabbit vessels or, at least, more prominent in rabbit retinal vessels. Second, dual-wavelength oximetry, fixed multi-wavelength oximetry, fixed-wavelength laser confocal scanning ophthalmoscopes, or hyperspectral slit-imagers are likely to underestimate such gradients due to limited spectral bandwidth, limited field-of-view and vessel segment averaging. We did not perform vessel segment averaging in our pseudocolored oximetry images. A previous hyperspectral study based on a slit-scanner suggests prominent intravascular oxygen gradients across the width of human retinal arteries and veins using second order polynomial approximation of vascular oxygen content [40]. Similarly, large longitudinal and transmural gradients of oxygen partial pressure have been demonstrated by microelectrode recordings along and within mucosal vessels [41]. Better understanding of these gradients using methods that allow their visualization over a large field is likely to provide more useful information about local tissue metabolism and is applicable to any tissue.

We performed multiple analyses to evaluate the contribution of spectral noise to our data. *In vitro* reflectance spectroscopy of whole blood and Monte Carlo simulations have suggested that scattered light originating from areas outside the vessel may contaminate vessel oxygen saturation measurements [42]–[44]. Therefore, use of dual-wavelength oximetry has required calibration for vessel size, fundus pigmentation and other unknown factors using linear calibration parameters [11], [15], [18]–[20]. Using our hyperspectral data set, we have adopted a different approach. We have empirically defined the collective spectral noise contribution for each pixel in every image by comparing the difference between the least-squares approximation of vessel optical density and the raw optical density measurements. This spectral noise index represents an average percent error for the reflectance measurements over all wavelengths. Where the error was higher than 5% suggests that wavelength data may be disproportionately confounded due to scatter or other illumination variability. These pixels constituted a very small fraction of all the pixels from a given scene (see Figure 7). In addition, the size and distribution of intravascular hemoglobin oxygen gradients does not uniquely correspond to areas of higher SNI, suggesting no correlation between the two. The validity of our algorithms is also supported by the experimental finding that HypS_ox_ measurements in pigmented and albino rabbits were similar and reproducible (Table 1).

In conclusion, hyperspectral computed tomographic imaging spectroscopy can overcome some of the limitations in spectral, spatial and temporal coregistration and resolution that have confounded spectroscopy *in vivo*. This system can be adopted to any imaging system and relies on standard image acquisition software and CCD cameras. While we have demonstrated proof-of-principal using hemoglobin oxygen saturation, investigators can study any biological target with spectral properties *in vivo* or *in vitro* using this method adapted to the appropriate imaging system. The results of our current experiments suggest that application of this method to other vascular tissues may reveal novel patterns of tissue oxygenation and vascular physiology.
